# Considerations for a Reliable In Vitro Model of Chemotherapy-Induced Peripheral Neuropathy

**DOI:** 10.3390/toxics9110300

**Published:** 2021-11-11

**Authors:** Sandy Eldridge, Arianna Scuteri, Eugenia M. C. Jones, Guido Cavaletti, Liang Guo, Elizabeth Glaze

**Affiliations:** 1 Division of Cancer Treatment and Diagnosis, National Cancer Institute, Bethesda, MD 20892, USA; glazee@mail.nih.gov; 2 Experimental Neurology Unit and Milan Center for Neuroscience, School of Medicine and Surgery, Milano-Bicocca University, 20900 Monza, Italy; arianna.scuteri@unimib.it (A.S.); guido.cavaletti@unimib.it (G.C.); 3 Fujifilm Cellular Dynamics, Inc., Madison, WI 53711, USA; eugenia.jones@fujifilm.com; 4 Investigative Toxicology Laboratory, Frederick National Laboratory for Cancer Research, Frederick, MD 21702, USA; liang.guo@nih.gov or; 5 Bristol Myers Squib, Princeton, NJ 08540, USA

**Keywords:** chemotherapy-induced peripheral neuropathy (CIPN), dorsal root ganglion (DRG), peripheral neurons, sensory neurons, Schwann cells, human-induced pluripotent stem cells (hiPSC), neurotoxicity, axonal degeneration, in vitro cell models

## Abstract

Chemotherapy-induced peripheral neuropathy (CIPN) is widely recognized as a potentially severe toxicity that often leads to dose reduction or discontinuation of cancer treatment. Symptoms may persist despite discontinuation of chemotherapy and quality of life can be severely compromised. The clinical symptoms of CIPN, and the cellular and molecular targets involved in CIPN, are just as diverse as the wide variety of anticancer agents that cause peripheral neurotoxicity. There is an urgent need for extensive molecular and functional investigations aimed at understanding the mechanisms of CIPN. Furthermore, a reliable human cell culture system that recapitulates the diversity of neuronal modalities found in vivo and the pathophysiological changes that underlie CIPN would serve to advance the understanding of the pathogenesis of CIPN. The demonstration of experimental reproducibility in a human peripheral neuronal cell system will increase confidence that such an in vitro model is clinically useful, ultimately resulting in deeper exploration for the prevention and treatment of CIPN. Herein, we review current in vitro models with a focus on key characteristics and attributes desirable for an ideal human cell culture model relevant for CIPN investigations.

## 1. Introduction

Chemotherapy-induced peripheral neuropathy (CIPN) is an adverse consequence of a wide variety of commonly used anticancer agents [1,2,3,4,5,6,7,8,9] and there are no gold standard therapeutics recommended for the prevention or treatment of CIPN [10]. CIPN frequently leads to dose reduction or discontinuation of therapy [4,11,12]. Clinical symptoms can persist long after completion of chemotherapy and severely diminish the quality of life of patients [1,13]. The pathophysiology of CIPN is complex and compounded by the fact that the various neurotoxic events culminating in CIPN are not necessarily related to the anticancer mechanisms of action for the agents that cause CIPN [9,14]. However, several lines of evidence point toward interactions involving various target components of the peripheral nervous system (PNS), including dorsal root ganglion (DRG), myelin, microtubules, mitochondria, ion channels, blood vessels, and nerve terminals [5,9,15,16,17,18]. A common pathology in CIPN is a “dying back” axon degeneration of distal nerve endings [9,19]. While it is not within the scope of this paper to review the pathogenesis of CIPN, readers are referred to several excellent and comprehensive reviews of the possible mechanisms involved in CIPN [8,16,20]. In this present paper, examples of agents that cause CIPN and can be used as tool compounds for the development of an in vitro model are provided to highlight the key features required for an in vitro cell model system designed to interrogate the pathogenesis of CIPN. Mechanistic understanding of axonal degeneration will provide insights into molecular pathways responsible for CIPN [9]. Development and appropriate use of cell-based models that recapitulate morphological and molecular features of peripheral neuropathy and application of relevant endpoint measurements will contribute greatly to understanding the pathogenesis of the disease [9,21,22]. This review aims to focus on key characteristics and attributes desirable for an ideal human cell culture model of CIPN for mechanistic explorations needed to elucidate the underlying pathophysiology and find effective treatments for CIPN.

## 2. Anticancer Agents That Cause CIPN

CIPN is a debilitating adverse effect with a prevalence ranging from 19% to over 85% [8] and caused by a spectrum of classes of widely used anticancer therapeutics including platinum-based agents, microtubule disruptors (taxanes and vinca alkaloids), proteasome, and angiogenesis inhibitors (Table 1) [1,2,3,4,5,7,8,9,23,24,25]. Clinically, CIPN symptoms may be acute, worsen with cumulative drug dosing, or emerge late during the course of treatment, even long after cessation of treatment [18]. Although many genetic and clinical risk factors have been identified, CIPN surveillance during and post-chemotherapy is needed as well as further study to better understand the pathophysiology of CIPN [8,18].

Among commonly used classes of cancer therapies for many blood and solid tumors, platinum analogs (e.g., cisplatin and oxaliplatin), proteasome inhibitors (e.g., bortezomib), immunomodulatory/antiangiogenic (e.g., thalidomide), and taxanes (e.g., paclitaxel) have markedly different chemical structures and mechanisms of actions. However, they all share a common adverse side effect: CIPN [16]. Clinically, CIPN involves the PNS that predominately leads to sensory axonal peripheral neuropathy characterized by a “stocking and glove” distribution of a plethora of potentially debilitating sensory effects [5,17]. Although the proposed pathogenesis of CIPN involves the cell bodies of the DRG concomitant with dying back axonal damage, the exact pathophysiology remains elusive [19,25]. Evidence suggests that neurotoxic chemotherapy drugs may involve various cellular components in the PNS by (1) forming DNA adducts, DNA damage, and alterations in DNA repair; (2) stabilization/disruption of microtubules; (3) targeting mitochondria; (4) functionally impairing ion channels; (5) production of oxidative stress; (6) dysregulation of calcium signaling; (7) altering cell signaling events; and/or (8) triggering immunological mechanisms through activation of satellite glial cells [9,14,15,16,17,18]. Development of an in vitro human peripheral neuronal cell model in which these various cellular components can be investigated will provide an urgently needed tool to dissect the cellular and molecular effects of potentially neurotoxic compounds.

The DRG of the PNS is vulnerable to neurotoxic damage since it is less protected by the blood–nerve barrier than the CNS [26,27]. This may partially explain the predominance of sensory involvement in patients with CIPN [9]. Platinum compounds form DNA adducts that can accumulate in the DRG [28,29], potentially leading to sensory neuronal cell death [30,31]. Paclitaxel has been reported to accumulate in the DRG through transmembrane transport mediated specifically by organic anion transporting polypeptide 1B (OATP1B) [32].

Central to the transport of proteins from the nerve cell body, down the length of the axon are microtubules [3]. A commonly used class of anticancer agents, taxanes, are microtubule binding agents, which produce polymerization that interferes with normal microtubule dynamics linked to disruption of axonal transport [24,33,34]. Another class of chemotherapy agents that cause CIPN are vinca alkaloids (vincristine, vinblastine, vinorelbine, and vinderine) that also bind tubulin and inhibit microtubule dynamics, leading to interference with the mitotic spindle [35]. A proteasome inhibitor, bortezomib, also affects microtubule polymerization independent of its mechanism as an anticancer agent [9,36].

Damage to the mitochondria that impairs mitochondrial function may play a pivotal role in CIPN [9,37]. For example, paclitaxel has been reported to cause functional impairment in axonal mitochondria [38]. Additionally, bortezomib has also been shown to cause accumulation of ubiquitin-conjugated proteins, mitochondrial dysfunction in peripheral sensory neurons (PNS) including Schwann cells, and endoplasmic reticulum stress particularly in Schwann cells [9,39,40,41].

There is reported evidence for direct toxicity to the distal axon terminals associated with peripheral neuropathy following cancer treatment with paclitaxel [42], thalidomide [43], and vincristine [44]. Oxaliplatin may affect the function of voltage-gated sodium (Na^+^) ion channels, inducing an acute peripheral neuropathy manifested by hyperexcitability [45,46,47].

Thalidomide is also associated with peripheral neuropathy through different proposed mechanisms [48]. Thalidomide-induced peripheral neuropathy is proposed to be mediated by its antiangiogenic effects [49]. Notably, attempts to establish a thalidomide rodent model both in vivo and in vitro have not been successful [9,50,51].

Although the underlying mechanisms responsible for the development of CIPN remain elusive and are further complicated by the diversity of anticancer agents that cause CIPN, there may be common degenerative pathways triggered when normal cellular processes and energy delivery mechanisms of the PNS become disrupted [9]. It is important to appreciate that mechanisms of CIPN may be shared by different classes of chemotherapeutic agents independent of their anticancer properties [3,4,5,6,7,8,9,12]. Some pathways, such as those activated by the mitogen-activated protein kinase (MAPK) family members and by mechanistic target of rapamycin (mTOR), could represent a common core of CIPN pathophysiology, as they have been demonstrated to be closely related to hyperalgesia and more in general to pain, a hallmark of CIPN [52,53,54,55,56]. The activation of MAPKs, and in particular of p38, has been observed to have a pivotal role in CIPN induced by different chemotherapeutic agents, such as paclitaxel, oxaliplatin, cisplatin, and vincristine [53,54,55,56] through their relation with toll-like receptor (TLR4) and nuclear factor kappa B (NF-kB) signaling pathways, not only in DRG neurons, but also in glial cells [52,53].

## 3. In Vitro Models

Development of fit-for-purpose in vitro models can contribute greatly to understanding the pathogenesis of CIPN and identifying intervention strategies [9,11,12,50,51,57,58,59,60]. Initially, the use of animal models enabled identifying the histopathological hallmarks of CIPN [61], but the need for a screening tool that is robust and reproducible highlights the necessity of simpler, faster, less expensive, and more relevant human models. This is also consistent with the principles of the 3Rs (Replacement, Reduction, and Refinement) in animal use in scientific research and testing [62].

The development of valid pre-clinical models of CIPN has been driven by the necessity to increase the knowledge of the pathogenic mechanisms of the different chemotherapeutic drugs, design more effective therapeutic strategies, screen for potential neurotoxicity of new drugs so as to inform patients of potential risks, and identify putative neuroprotective compounds [9].

Unfortunately, the clinical complexity of CIPN adds to the challenge of developing a unique and effective human in vitro model for CIPN. The clinical condition of CIPN is actually the sum of different pathological features shared by several antineoplastic drugs; however, these features may also differ according to antineoplastic drug class [14]. For this reason, the selection of the in vitro model should always consider the drugs being used in determining fit-for-purpose [57].

Over the years, different kinds of in vitro models have been developed, with an increasing complexity and informative degree. Initially, cell cultures were used for this purpose, starting from neuronal-like cell lines, such as PC-12 and SH-SY5Y, differentiated into a more mature neuronal phenotype using agents such as nerve growth factor and retinoic acid [57]. Such models have been followed by primary and/or organotypic culture of DRG, the focus target of chemotherapeutic drugs. The DRG is a cluster of neurons located adjacent to the spinal cord [22]. The DRG are comprised of a heterogeneous population of PSN derived from neural crest cells [22]. Primary rodent DRG explants or dissociated rodent DRG cell cultures have been used to recapitulate the pathophysiological features of peripheral neuropathy and axonal degeneration and to elucidate underlying mechanisms of CIPN [3,11,50,63,64].

DRGs are obtained from healthy mice or rats and cultured as organoids or dissociated to obtain primary cultures for testing neurotoxic as well as neuroprotective agents in vitro. Additionally, DRGs may be directly explanted from animals previously treated with test agents to evaluate drug effects on DRGs in situ. Both preclinical models have been used to elucidate mechanisms of CIPN [57]. As stated earlier, unprotected by the blood brain barrier (BBB), DRGs are more easily reached by drugs, and, at this level, relevant alterations have been observed in response to agents that cause CIPN [65]. One of the most notable effects of neurotoxic drugs in DRG in vitro models is the reduction of neurite length. DRG organotypic cultures have been mainly used as a neurotoxicity-screening model to evaluate the effect of the different drugs on the neurite elongation [66,67,68]. DRG organotypic and DRG dissociated primary cultures have been used to explore molecular mechanisms involved in the development of CIPN and find neuroprotective targets [69].

Primary cultures are limited by an inherent lack of reproducibility and standardization [70]. Furthermore, rodent systems are not an ideal substitute for a cell model of human origin. The lack of accessibility of human neural tissue (from biopsies or post-mortem) combined with challenges in culturing neuronal cells from these sources has resulted in only a limited contribution of human tissues in the understanding of the pathophysiology of CIPN to date.

Newer and more informative models have been created with the development of mixed co-cultures and with an emerging approach to create a 3D model by exploiting the Matrigel substrate [71,72,73]. The production of organoids from human induced pluripotent stem cells (hiPSCs) for developing functionally relevant in vitro models of the human brain and DRG is an area of active research [74,75]. With recent advances in microfluidic and confocal microscopic technologies, analysis of axonal neuropathy may be performed in the peripheral neural microphysiological system [72]. This 3D ‘nerve-on-chip’ model recapitulates the complexity of myelinated human peripheral nerves in vivo and allows for in vitro evaluation of nerve conduction velocity and neurite volume as potential endpoint measurements to assess damage in sensory axons. The technology is labor intensive, lacks reproducibility, and does not allow scaling at this point in early development [76,77]. It is not currently possible to create standardized organoids with a specific and reproducible cellular composition of mature native nervous tissue with electrophysiological activity [78,79].

Finally, CIPN is one of the most common adverse events of many antineoplastic drugs, but not all patients receiving therapy develop it, or to the same degree. There is indeed a genetic component, which makes the situation even more complex [80,81]. The latest frontier of in vitro model design is moving in that direction, with the use of patient-derived hiPSCs [82]. Such cell culture models would allow deriving differentiated neurons starting from somatic cells easily obtained from the patient (e.g., peripheral blood mononuclear cells or fibroblasts) for a personalized medicine approach that considers the genetic component.

Advances in stem cell biology to differentiate human embryonic stem cells (hESCs) or hiPSCs into PSN offer a promising technology to provide novel human-based, clinically relevant, in vitro models to study not only CIPN, but also PSN development and injury in general [22,83,84,85,86,87,88,89,90,91]. In particular, hiPSCs offer unique potential for reliable and robust applications for disease modeling and drug screening [92]. Their capacity for self-renewal and widely diverse differentiation, coupled with the ability to generate large quantities of specific cell types in a controlled and reproducible manner, make them ideal for high throughput screening platforms to discover and evaluate safety and efficacy of novel therapeutics [92].

A list of commercially available hiPSC-derived neurons (CNS and PNS) is provided in Table 2 and is not intended to be exhaustive given the rapidly evolving market for such cells. Unfortunately, to the best of our knowledge, no commercial vendors supply mature hiPSC-peripheral neurons as a differentiated ready-to-use, readily available product. Ncardia’s Peri4U™ human product had been available and successfully used to demonstrate sensitivity to CIPN agents [86,91]. However, Ncardia discontinued its commercial Peri4U™ product and instead offers a custom-ordered hiPSC-peripheral neuronal cell model that is not readily available. Some commercial vendors offer undifferentiated neural crest cells, but these cells require extensive expertise to maintain reproducibility and readily available cultures [93]. Furthermore, differences in seeding conditions, pluripotency status of hiPSCs prior to differentiation, and in the differentiation and maturation protocol can significantly alter the composition of the desired cell model [93]. Additionally, lack of in-depth phenotypic and functional characterization is a common drawback of all hiPSC-peripheral neuronal products currently available in the commercial market.

The application of hiPSC-derived CNS neurons to assess CIPN-inducing agents has been evaluated by several laboratories using commercially available cells; although results support the use of hiPSC CNS cells for toxicity testing of some compounds with CIPN liability [59,88,89,91,94,95,96], CNS cells do not recapitulate PNS cell susceptibility to CIPN. Therefore, the use of mature human PSN cell model is highly desirable to serve the global research community.

A reliable source of human PSN is essential (1) to develop a better understanding of the mechanisms involved in developing peripheral neuropathies, (2) to drive the development of next generation chemotherapeutic drugs and treatment regimes, and (3) for the development of therapeutic agents to mitigate CIPN.

## 4. Outcome Measurements in an Ideal Human Peripheral Sensory Neuronal Cell Model

### 4.1. Morphologic Features

Anatomically, peripheral neurons are pseudo-unipolar with cell bodies located in the DRG of the spinal cord and axons ending in the spinal cord and peripheral branches throughout the body [97]. Although the fundamental mechanisms of CIPN are not completely understood, a major pathology in this syndrome is a “dying back” phenomenon, referred to as Wallerian degeneration, characterized by axonal degeneration of distal nerve endings [19]. An understanding of the mechanistic basis of axonal degeneration will help to reveal the pathways and molecular dynamics responsible for CIPN. The application of models that recapitulate essential morphologic and functional features of peripheral neuropathy and apply fit-for-purpose endpoint measurements will contribute to understanding the pathogenesis of CIPN [21].

It seems reasonable to expect a hiPSC-derived PSN cell model will serve as an in vitro cell-based system that is sensitive to agents that may induce peripheral neuropathy and to screen for potential therapeutic modalities that may prevent or reverse the adverse effects of CIPN agents. The cellular measurements that are relevant to the human disease include neuronal cell viability, axon outgrowth, and degeneration in terms of neurite length, width, and area, and branching of axons that reflect the “dying back” axon degeneration observed clinically [9,57,89].

### 4.2. Gene Expression

The human PNS is a complex network of functionally distinct neurons responsible for sending and receiving transduction signals throughout the body [22]. Terminally differentiated sensory neurons are classified based on their innervation targets, neurotransmitter synthesis profiles, axon diameter, myelination status, neurotrophic factor dependency, and corresponding neurotrophic tyrosine receptor kinase (NTRK) expression signatures [22,98,99]. Peripheral sensory neurons (PSN) receive and transmit external stimuli to the spinal cord. The types of signals PSN transmit to the CNS include pain (nociception), touch (mechano-sensation), temperature (thermoception), and balance and position (proprioception) [97].

The transduction pathways for electrical currents in the PNS include genes for transient receptor potential channels (TRP), sodium channels (e.g., Nav1.8 and Nav1.9), potassium channels (e.g., TRAAK and TREK-1), and a variety of voltage-gated calcium channels [98]. An example of drug-induced increases in neuronal excitability and changes in gene expression of select neuronal ion channels in DRG comes from a study of paclitaxel-treated rats that provides insight into the molecular and cellular basis of paclitaxel-induced peripheral neuropathy [100].

To create a cell product substitute for neurons isolated from DRG, a human iPSC-derived PSN cell model is highly desirable. Next-generation transcriptomics has been applied to demonstrate differential expression between mouse and human DRGs in a variety of mRNAs for transient receptor potential channels, cholinergic receptors, potassium channels, sodium channels, and other markers/targets [101]. Using DRGs obtained from patients with and without neuropathic pain, electrophysiology and paired gene expression profiling revealed key contributing gene modules and signaling pathways related to neuropathic pain that may lead to therapeutic strategies [102]. Readers are referred to the full transcriptomic dataset and code for analysis that the authors have kindly made available at: https://www.utdallas.edu/bbs/painneurosciencelab/sensoryomics/hdrgclinical/ (accessed on 11 November 2021) [102].

### 4.3. Protein Expression

The most commonly used protein expression markers used in cell culture models of peripheral neurons include NeuN, a protein expressed exclusively in neuronal cell nuclei, and Tuj-1, also known as class III β-tubulin, a component of neuron-specific tubulin [22,50,57] (Table 3). Vimentin is a type III intermediate filament protein expressed in mesenchymal cells [103]. It is a non-specific biomarker used to stain cell bodies and processes of non-neuronal cells [50], compared to S100 that is specific for Schwann cells [104].

### 4.4. Functional Analysis

Besides the in vitro model used, it is also critically important to evaluate the kind of analyses performed: sometimes the problem is not the model, but the way assays are performed [105]. Each drug has its own mechanism of action and neurotoxicity, which should be considered. Some mainly affect the neuronal soma, whereas others have axons as main target. For example, vincristine affects electrophysiological properties of axons, but not of the soma [106]. A functional real-time assay has come from the development of the multi-electrode array (MEA). The MEA instrument provides real-time monitoring of spontaneous electrophysiological activity within in vitro neuronal cell cultures [107], including multiple cell types present in a DRG cell system [108]. Multi-well MEAs provide a functional platform for assessing compound-related effects combining a label-free, electrophysiological readout with the ability to multiplex with other relevant endpoints.

While MEA assays analyze functionality of neuronal cell cultures at the multicellular level, both patch clamp and intracellular Ca^2+^ measurement enable characterizing expression of functional receptors and ionic channels responsible for sensory processing at the single cell level [109,110]. Further, measurement of axonal transport function through labelled mitochondria can provide mechanistic insight of CIPN for some anticancer drugs such as paclitaxel [111].

Several authors have observed hyperexcitability in DRG neurons, due to alterations in sodium and potassium channels. In particular, Li and colleagues demonstrated the increased expression of the voltage-gated sodium channel 1.7 (Nav1.7) in those DRGs most commonly involved in CIPN after paclitaxel exposure [112]. More recently, other authors, using both mathematical and cell-based models exposed to several drugs, suggested that different channels may be involved also, such as sodium channel Nav1.8 as well as delayed rectifier potassium channels [113], and hyperpolarization-activated cyclic nucleotide-gated (HCN) channels [114].

Besides the electrophysiological alterations, studies have focused attention on the inflammatory response triggered by chemotherapeutic drugs on DRG neurons. Zhang and colleagues evidenced the activation of TLR4 and monocyte chemoattractant protein 1 (MCP-1) in DRG after paclitaxel exposure, with a consequent macrophage infiltration [115]. Macrophages were shown to activate an inflammatory response through the involvement of different molecular pathways, such as those of MAPKs [53] and calcineurin/nuclear factor-activated T cells (NFAT) [116]. This led to hypersensitivity and ultimately manifested as a measurable loss of intraepidermal nerve fibers

Another fundamental target of CIPN-induced alterations that has been identified in DRG-based models is mitochondria. Several authors have reported a reduction in mitochondrial bioenergetics and motility in DRG models [117,118]. This effect may be due to alterations in DRG neuronal cytoskeleton, often the target of the toxic effect of chemotherapeutic compounds, but also to cytokine production (such as IL-10) triggered by some CIPN agents [117,118].

All these aspects should be considered during the translation process towards a novel experimental model, such as hiPSCs, because we must ensure that these ad hoc cellular models possess the pivotal pathways identified in historical models.

### 4.5. High-Content Analysis

CIPN is predominantly a sensory neuropathy that is histopathologically characterized by “dying back” axonal degeneration that proceeds in a distal-to-proximal manner [19,57]. Image analysis of neurite outgrowth, therefore, is commonly used to quantify morphological alterations caused by CIPN-inducing agents [11,19,50,59,66,85,86,88,91]. Measurement of neurite length is frequently used to investigate axon degeneration in vitro [57]. Typically, fixed cell cultures are immunohistochemically stained with a neuronal marker, such as Tuj-1 (βIII-tubulin). Neurite length, area, and width are measured by the use of image acquisition platforms with image analysis software [50,57]. In addition, branching of neurites has been shown to be reflective of axonal damage by some CIPN-causing agents such as paclitaxel [72,86]. By multiplexing biomarkers of key attributes in cell cultures, including neurons, neurites, mitochondria, intermediate filaments, etc., a plethora of measurements can be made using high content image analysis software and analyzed for drug effects [50]. Therefore, it is critical that an in vitro cell model be amenable to such analyses that include neurite area, neurite branchpoints, neuronal cell number, cell viability, apoptosis, mitochondrial impairment, electrophysiological effects, etc. A multiparametric analysis system can detect all the changes in a cell culture model and therefore is able to discriminate drug-specific variations [50].

With recent advances in microfluidic and microscopy technologies, analysis of axonal neuropathy can now be performed in the peripheral neural microphysiological system [72]. This 3D nerve-on-chip model recapitulates the complexity of myelinated human peripheral nerves and allows use of neurite volumes as a potential endpoint measurement to assess damage in sensory axons.

### 4.6. Scalability, Sensitivity, Specificity and Reproducibility

An in vitro cellular model based on recapitulating the human PNS has the potential to greatly enhance the understanding of CIPN and contribute to development of effective therapies. Highly efficient and reproducible protocols for differentiation of hiPSCs into different types of peripheral neural cells is one requirement that is not trivial [119]. Furthermore, to meet the needs of a widely dispersed research community, production of the model system must be scalable. It is anticipated that scalable multi-well plate and automated systems will be used for mechanistic assays for investigative studies, disease modeling, drug screening and drug development. It will be critical to qualify the cell model and applied assays to ensure that accurate and precise results are obtained from these systems. Part of the qualification will be assessment of sensitivity and specificity using proper positive and negative control agents appropriate for each assay being applied. While there are many aspects to these studies that may result in variability issues, investigators will need to prepare rigorously defined protocols to test experimental conditions that contribute to sources of variability, including, for example, plate preparation, cell characterization, culture conditions, media, cell plating density, stability, etc., to ensure consistent results [120]. To obtain a standard, functionally active human iPSC-derived PNS model system, an integrated approach is required to fulfill the unmet needs of the scientific community engaged in PNS research.

## 5. Conclusions

Cell culture models serve as a valuable tool for mechanistic studies and drug screening strategies with appropriate outcome measures using functional, molecular, and biochemical methods. The application of stem cell technologies to generate cell type characteristic of the PNS is a promising approach to developing a human-relevant and translational model system. Here, we have described desirable components of an “ideal” cell model in terms of morphologic features, gene expression, protein expression, functionality, high content imaging, scalability, and performance in terms of sensitivity, specificity, and reproducibility. Key attributes of an ”ideal” human cell-based system to model CIPN, screen novel therapies for CIPN activity, and explore preventive and treatment strategies are listed in Table 4.

The application of hiPSCs to generate a variety of cell types found in the peripheral nervous system presents an exciting opportunity to explore the interactions of these various cell types (Schwann cells, satellite glial cells) in a controlled fashion. Advancements in understanding the role of Schwann cells in the pathology of neuronal disease, including peripheral neuropathy [121,122,123,124,125,126,127], highlight their consideration for development of more complex in vitro models of CIPN. In the meantime, a hiPSC-derived PSN cell model is urgently needed to advance the understanding of the pathogenesis of CIPN and identify gaps requiring deeper exploration for the prevention and treatment of CIPN.

## Figures and Tables

**Table 1:** Anticancer agents known to cause CIPN and their proposed mechanisms and target sites of CIPN toxicity [1,2,3,4,5,7,8,9,23,24,25].

Class	Agents	Proposed Mechanism	Main Target of CIPN Toxicity
Taxanes	Paclitaxel Docetaxel Ixabepilone	Microtubule disruption	Dorsal root ganglion; axons; distal nerve terminals
Platinum-based	Cisplatin Carboplatin Oxaliplatin	DNA adducts	Dorsal root ganglion
Alkylating agents	Cyclophosphamide Hexamethylmelamine Ifosphamide Procarbazine	Covalently bind to DNA	Dorsal root ganglion
Vinca alkaloids	Vincristine Vinblastine Vinorelbine Vindesine	Dysfunction of mitochondria and endoplasmic reticulum; microtubule disruption	Dorsal root ganglion; distal nerve terminals
Proteasome inhibitors	Bortezomib Carfilzomib Ixazomib	Binds proteasome complex; mitochondrial disturbance; microtubule disruption	Dorsal root ganglion and peripheral nerves
Immunomodulatory	Thalidomide Lenalidomide Pomalidomide	Antiangiogenesis	Dorsal root ganglion; distal nerve terminals

**Table 2:** List of vendors offering hiPSC-derived cells for neurotoxicity research.

Vendor	Cell Product
Anatomic ^1^, Minneapolis, Minnesota, US	Human iPSC- RealDRG^TM^ Nociceptors ^2^
Applied StemCell, Milpitas, California, US	Human IPCS Sensory Neuron Differentiation ^3^
Axol, Cambridge, United Kingdom	Human iPSC-Sensory Neuron Progenitors ^4^
BrainXell, Baltimore, Maryland, US	Human iPSC CNS Neurons ^5^
FCDI ^6^, Madison, Wisconsin, US	Human iPSC iCell Dopa Neurons Human iPSC iCell GABA Neurons Human iPSC iCell Motor Neurons
iXCells Biotechnology, San Diego, California, US	Human iPSC Motor Neurons Human iPSC Cortical GABAergic Neurons Human iPSC Dopaminergic Neurons
Ncardia, Leiden, Netherlands	Human iPSC Peripheral Neurons ^7^
Nexel, Seoul, Korea	Neurosight^®^—Human iPSC Neurons ^8^
NeuCyte, San Carlos, California, US	SynFire^®^—Human iPSC CNS Neurons ^9^
REPROCELL, Yokohama, Japan	StemRNA™ Neuro—Human iPSC Brain Neurons
Takara Bio, Inc., Shiga, Japan	Human Neural Stem Cells ^10^

^1^ Small start-up company working out of University of Minnesota incubator; ^2^ Requires further maturation in culture for one week; ^3^ Offers differentiation services; ^4^ Requires maturation in culture for at least 3 weeks; ^5^ Directed differentiation protocol can generate subtype-specific CNS neurons and glial cells; ^6^ Fujifilm Cellular Dynamics, Inc.; ^7^ Custom orders can result in long lead time (≥2 months); ^8^ Type of neurons not specified; intended use is functional electrical activity; ^9^ Early-stage biotechnology company with proprietary differentiation protocol that can generate multiple neuronal cell types from the CNS that may be co-cultured with glial cells; focus is on CNS diseases; ^10^ Requires extensive differentiation protocol by end-user.

**Table 3:** Commonly used protein expression markers of differentiated peripheral sensory neurons [22].

**Protein Marker**	**Function**
βIII-tubulin (Tuj-1)	Tubulin beta III (TUBB3), also called Tuj-1, is a structural protein of the cytoskeleton in neurons
Peripherin	Type III intermediate filament protein expressed mainly in neurons of the PNS
NeuN	Neuronal specific nuclear protein found in both the CNS and PNS
MAP2	Microtubule-associated protein 2 neuron-specific cytoskeletal protein
BRN3A	POU * transcription factor expressed in the DRG

* The acronym, POU, is derived from the names of three transcription factors: (1) the Pituitary-specific Pit-1, (2) the Octomer-binding proteins Oct-1 and Oct-1, and (3) the neural Unc-86 transcription factor from *Caenorhabditis elegans*.

**Table 4:** Attributes of an “ideal” cell-based system to model CIPN.

Category	Attributes
Components of the PNS to model either as pure nociceptor neuronal cultures or co-cultures to include multiple cell types	Human-induced pluripotent stem cell origin Peripheral sensory neurons with axons Dorsal root ganglion with pseudo-unipolar neurons Satellite glial cells Schwann cells Endothelial cells
Production of cells	Cells must be scalable, highly reproducible, high purity, high viability (~80%) upon thawing
Protein biomarker examples (see Table 1)	NeuN—Neuronal-specific nuclear protein Tuj-1—βIII-tubulin structural protein of the cytoskeleton in neurons BRN3A—transcription factor expressed in the DRG SOX10—transcription factor expressed in Schwann cells
Cell culture format	Accommodate multiple plate formats, e.g., 6-well, 12-well, 96-well, and 384-well Ready for use within a few days of plating Must remain viable long enough to allow for short-term (5–7 days) or long-term (4–6 weeks) cultures
Live cell assays	Must be able to perform live cell assays such as those incorporating dyes for determining viability (live and dead cells), mitochondrial function, mitochondrial motility, calcium transients
Structural measurements	Must have pathology similar to human CIPN pathology so as to recapitulate clinical morphologic changes in response to CIPN agents Cells must be able to withstand fixation and immunostaining procedures without compromising morphologic integrity in multiplexed assays
Functional measurements	Cells must be amenable to multi-electrode array real-time monitoring of spontaneous electrophysiological activity

## Data Availability

Not applicable.
